# Real-time three-dimensional MRI for the assessment of dynamic carpal instability

**DOI:** 10.1371/journal.pone.0222704

**Published:** 2019-09-19

**Authors:** Calvin B. Shaw, Brent H. Foster, Marissa Borgese, Robert D. Boutin, Cyrus Bateni, Pattira Boonsri, Christopher O. Bayne, Robert M. Szabo, Krishna S. Nayak, Abhijit J. Chaudhari

**Affiliations:** 1 Department of Radiology, University of California Davis, Sacramento, California, United States of America; 2 Department of Biomedical Engineering, University of California Davis, Davis, California, United States of America; 3 Department of Orthopaedic Surgery, University of California Davis, Sacramento, California, United States of America; 4 Ming Hsieh Department of Electrical Engineering, University of Southern California, Los Angeles, California, United States of America; Virginia Tech, UNITED STATES

## Abstract

**Background:**

Carpal instability is defined as a condition where wrist motion and/or loading creates mechanical dysfunction, resulting in weakness, pain and decreased function. When conventional methods do not identify the instability patterns, yet clinical signs of instability exist, the diagnosis of *dynamic instability* is often suggested to describe carpal derangement manifested only during the wrist’s active motion or stress. We addressed the question: can advanced MRI techniques provide quantitative means to evaluate dynamic carpal instability and supplement standard static MRI acquisition? Our objectives were to (i) develop a real-time, three-dimensional MRI method to image the carpal joints during their active, uninterrupted motion; and (ii) demonstrate feasibility of the method for assessing metrics relevant to dynamic carpal instability, thus overcoming limitations of standard MRI.

**Methods:**

Twenty wrists (bilateral wrists of ten healthy participants) were scanned during radial-ulnar deviation and clenched-fist maneuvers. Images resulting from two real-time MRI pulse sequences, four sparse data-acquisition schemes, and three constrained image reconstruction techniques were compared. Image quality was assessed via blinded scoring by three radiologists and quantitative imaging metrics.

**Results:**

Real-time MRI data-acquisition employing sparse radial sampling with a gradient-recalled-echo acquisition and constrained iterative reconstruction appeared to provide a practical tradeoff between imaging speed (temporal resolution up to 135 ms per slice) and image quality. The method effectively reduced streaking artifacts arising from data undersampling and enabled the derivation of quantitative measures pertinent to evaluating dynamic carpal instability.

**Conclusion:**

This study demonstrates that real-time, three-dimensional MRI of the moving wrist is feasible and may be useful for the evaluation of dynamic carpal instability.

## Introduction

The wrist is comprised of a complex arrangement of carpal bones, ligaments, and tendons. These tissues collectively maintain biomechanical stability during the wrist’s physiologic motion and loading[1–5]. When wrist ligaments are injured (e.g., owing to a fall or other excessive load on the wrist), the normal anatomical alignment of the carpal bones can be disrupted[6–8], with consequent pain, dysfunction, and premature osteoarthritis[9]. The resulting joint instability will typically present along a spectrum from “dynamic instability” (revealed only when the wrist is in motion or under stress[10, 11]) to “static instability” (which can be visualized with standard imaging of the motionless wrist)[12]. To assess dynamic instability, methods capable of capturing carpal bone trajectories during active motion or dynamic loading are needed. If physicians can diagnose dynamic instability early, interventions can be implemented to restore normal wrist function[5, 10, 13, 14].

Magnetic Resonance Imaging (MRI) is the most commonly used tomographic imaging modality for evaluating wrist ligament derangements[15]. However, standard MRI evaluates the stationary, immobilized wrist and is unable to directly evaluate dynamic carpal instability. Only a few studies have reported the use of fast MRI acquisition to visualize carpal kinematics[11, 16, 17]. This is primarily due to the slow encoding of k-space inherent to MRI that results in low temporal resolution, and the presence of susceptibility and other artifacts that may compromise image quality[11]. Several advances have been made recently to improve the performance of MRI for real-time data-acquisition, such as using non-Cartesian sampling and/or sparse sampling coupled with constrained image reconstruction[18–22]. To date, these advances have been applied to investigate motion of the knee[16, 23, 24] and temporomandibular joint[25, 26] but not to carpal motion. There has been a concern that fast gradient-echo based pulse sequences that worked well for other joints may not be technically feasible in the moving wrist because magnetic field inhomogeneities generated by the significant displacement of the tissues during wrist motion may generate substantial artifacts[11]. Therefore, we sought to develop a method for imaging the moving wrist, that overcame this challenge.

Our overall goal was to optimize real-time three-dimensional (3D) MRI (data-acquisition and reconstruction) for imaging the unassisted, actively moving wrist and demonstrate the feasibility of deriving standardized imaging metrics relevant to evaluating dynamic carpal instability. The total acquisition time (multiple active maneuvers) was aimed to be quick (< 2 min, scan time for the wrist with motion) to have the ability to be incorporated into the current workflow of musculoskeletal radiology practice, as a supplement to a routine clinical MRI wrist scan. We utilized two real-time MRI pulse sequences, four sparse data-acquisition schemes, and three constrained image reconstruction methods, and assessed these techniques during the performance of wrist radial-ulnar deviation and the clenched-fist maneuvers.

## Materials and methods

### Study subjects and positioning on scanner bed

This study received approval from the University of California Davis Institutional Review Board (IRB). Written informed consent was obtained from all participants before study participation, based on the approved documentation and guidelines of the IRB. Two participants (both men) were recruited for imaging protocol development and optimization, while ten participants (6 men and 4 women) were recruited for evaluating the protocol. The average age of the participants was 27.6±6.1 years. Inclusion criteria included asymptomatic wrists, age <60 years, the ability to lie prone in the “superman position” during scanning, and the ability to follow directions to perform wrist motions while in the MRI scanner. Exclusion criteria were standard contraindications to MRI (including claustrophobia) and history of wrist pain and derangements (including trauma and arthritis).

Scanning was performed on a 3.0T MRI system (Skyra, Siemens Healthcare, Erlangen, Germany) using a 32-channel radiofrequency (RF) head-coil (to accommodate the large range-of-motion of the wrist during its different maneuvers). The participants lay on the scanner bed in the “superman position” with one arm out-stretched above the head into the RF coil (standard position employed for clinical wrist MRI acquisition). The wrist was placed into an arm immobilizer and each participant was trained to hold the wrist motionless in its neutral position for up to 7 minutes (for a static MRI acquisition) and next, perform the following two wrist maneuvers utilizing their full, active range-of-motion (absent pain) at a comfortable speed (i.e., continuously between the “start” and “stop” instruction interval of 20 s, completing at least 2 cycles of each maneuver): [i] radial-ulnar deviation, and [ii] the clenched-fist maneuver with the wrist in the neutral position. They were asked to repeat the two wrist maneuvers each 3 times to allow the assessment of test-retest reliability. Both wrists were scanned for each individual.

### Imaging protocol development and optimization

The developed protocol consisted of two sections optimized and evaluated based on phantom studies and scanning of the two participants. First, a gradient-recalled-echo (GRE)-based 3D pulse sequence (referred to by the scanner manufacturer as volumetric interpolated breath-hold examination (VIBE)) was optimized for high-spatial-resolution imaging (voxel size: 0.20 × 0.20 × 0.30 mm) of the immobilized wrist in the wrist’s neutral position. This scan served as our anatomical reference.

Next, two real-time MRI pulse sequences were implemented and optimized, namely (1) balanced steady-state free precession (bSSFP)-based, referred to by the scanner manufacturer as true fast imaging with steady state precession (TrueFISP), and (2) fast GRE-based, referred to by the scanner manufacturer as fast low angle shot (FLASH). The optimization was driven by determining a trade-off between the voxel size, bandwidth, slice thickness, field-of-view (FOV), and signal-to-noise-ratio (SNR), as described in subsequent paragraphs. Tissue contrast was chosen by tuning repetition time (TR), echo time (TE), and flip angle. Automated high-order B_0_ field shimming was performed while the wrist was held motionless in the neutral position before beginning the optimization procedures. Scan parameters were chosen by consensus between the authors.

The scan parameters for bSSFP were matched with those reported in Boutin et al[11]. A comprehensive comparison of bSSFP and fast GRE was made by including Cartesian (rectilinear) and radial k-space sampling schemes for each sequence. The key imaging parameters the authors converged on for both pulse sequences are given in Table 1. The other scan parameters common to both pulse sequences were field-of-view (FOV) of 120 mm^2^, acquisition matrix size of 112 x 112, in-plane resolution of 1.07 x 1.07 mm, and slice thickness of 6 mm, with 6 slices acquired in the coronal plane per volume using the default sum-of-squares-based image reconstruction.

**Table 1:** Imaging parameters used for fast-GRE and bSSFP pulse sequences for scanning the actively moving wrist (R-radial, C-Cartesian).

Sequence	R-Fast-GRE	C-Fast GRE	R-bSSFP	C-bSSFP
Temporal Resolution (ms)	315/194/134	351	476	477
Voxel Size (mm^3^)	1.07×1.07×6	1.07×1.07×6	1.07×1.07×6	1.07×1.07×6
Radial Spokes or Cartesian Lines	100/60/40	112	100	112
TR/TE (ms)	[3.15/1.94/1.34]/1.74	3.13/1.74	4.77/1.76	4.25/1.75
Bandwidth (Hz/pixel)	990	990	930	930
Flip Angle	12	12	46	46

We first confirmed that susceptibility/banding artifacts, that are unique to bSSFP sequences[11] partially obscured the wrist during motion. Although dielectric pads could provide an option to reduce banding, the pads limit range of motion[11] and therefore their use was avoided. The radially-sampled fast-GRE method provided a reasonable spatial resolution with lesser artifacts (spokes = 100) compared to bSSFP (discussed further in our Results); thus, we optimized the former further and analyzed the effect of voxel sizes and radial spokes on temporal resolution. Different in-plane spatial resolutions (by fixing the slice thickness to 6 mm), radial spokes (or angular undersampling), SNR, and their corresponding temporal resolutions were evaluated to determine suitable values relevant to this application. To satisfy the Nyquist criterion, 176 radial spokes are necessary to completely cover the k-space, (i.e., N_radial_ = $\left(\frac{\pi }{2}\right)$ × N_Cartesian_, where N = number of frequency encoding lines). However, the resulting temporal resolution for 176 radial spokes was 554 ms (or less than 2 frames per second [fps]). We therefore chose 100 spokes as a starting point to have a moderate temporal resolution of about 3 fps, and then progressively reduced to acquiring 60 spokes (5 fps) and 40 spokes (7 fps) spokes.

Based on these assessments, our scan time for the static scan was 6 min, 30 s, while scans for each dynamic maneuver lasted 19 s (100 spokes), 12 s (60 spokes), and 8 s (40 spokes), keeping the same number of acquired time points (total of 10). This developed protocol was then employed in the next phase involving the ten participants (twenty wrists). Additionally, each scan for the dynamic maneuvers was repeated thrice for each wrist for test-retest analysis. At the end of each wrist scan, the raw k-space data were obtained from the scanner console and reconstructed, as described in the next section.

### Constrained image reconstruction

Our approach to constrained image reconstruction using sparsity constraints was based on Lustig et al.[18]. Accordingly, our image reconstruction problem was posed as:

$$\hat{x}=\arg\;\underset{x}{\min}{\left|\left|Fx-y\right|\right|}_{2}^{2}+{\alpha ||Wx||}_{1}+\beta {\left||Dx|\right|}_{1},$$ [1]

where $\hat{x}$ is the reconstructed image, *F* and *y* are the Fourier encoding operator (undersampled) and the k-space measurements, respectively, *W* and *D* denote the discrete cosine transform (DCT) and finite-differences (FD) operator, respectively, and *α* and *β* are the regularization parameters that control the weighting for the two penalty functions. Sparsity is enforced by the choice of the $l_{1}$ norm for each of the penalty functions. The DCT and FD schemes were applied along the spatial dimension. In this framework, setting *α* = 0 or *β* = 0 results in only FD-sparsity or only DCT-sparsity-based minimization, respectively. The nonlinear conjugate gradient (CG) algorithm with backtracking line search was used to optimize the cost function in Eq (1). The gradient of the objective function was:

$$\nabla f(x)=2F^{*}(\mathrm{Fx}-y)+\alpha \nabla {\left|\left|Wx\right|\right|}_{1}+\beta \nabla {\left|\left|Dx\right|\right|}_{1},$$ [2]

The $l_{1}$-norm is the sum of absolute values, so the function is not smooth. Therefore, we approximated the $l_{1}$-norm as $|x|\approx \sqrt{x*x+\mu }$, with a smoothing parameter, μ ≪ 10^−16^. With this approximation, the derivative of the $l_{1}$-norm became $\frac{d|x|}{dx}\approx \frac{x}{\sqrt{x^{*}x+\mu }}$, which was used to compute the gradient of the cost function in Eq (1).

For the constrained reconstruction analysis, we compared the conventional gridding-based reconstruction, FD-sparsity (with α = 0), DCT-sparsity (with β = 0), and FD+DCT sparsity-based penalties. The non-linear CG algorithm was implemented using MATLAB (MathWorks, Natick, MA). Each channel’s data were individually reconstructed, and then the final image was combined by the root-sum-squares method. Details regarding our choice of the regularization parameters are provided in the next section. All four reconstruction methods were performed on a workstation with a 3.4 GHz Intel i7 processor and 128 GB RAM. Image Reconstruction Toolbox (IRT)[27] was used to perform the gridding-based reconstruction from radially sampled k-space data and to build the undersampled Fourier operator (F) to solve Eq (1).

### Parameter tuning and mathematical convergence

Choosing an appropriate regularization parameter (α or β in Eq (1)) is crucial for the suppression of undersampling artifacts in reconstruction. To compare regularization schemes, we adopted the L-curve approach, which eliminates the heuristic selection of the regularization parameter(s). This requires an iterative search by solving the minimization schemes with various regularization parameters and plotting the model error (${\left|\left|Fx-y\right|\right|}_{2}^{2}$) and prior error (||W(x)||_1_ or ||D(x)||_1_) to pick the elbow of the curve that denotes the optimal regularization parameter. Note that selection of α and β simultaneously for FD+DCT sparsity optimization is not straightforward using the L-curve as it requires determining the corner of the hypersurface[28]. Hence, both α and β were empirically chosen for this estimation. Further, we investigated the mathematical convergence behavior of the three types of penalties to evaluate the consequence of using more than one penalty in the cost function. The gridding reconstruction was set as the initial guess for all the three iterative reconstructions.

### Image quality assessment

Three fellowship-trained, board-certified musculoskeletal radiologists, one with 21 years of experience, second with 10 years of experience, and the third with 9 years of experience, participated in the human observer study. They first met with the researchers, finalized the viewing settings, and reached consensus on scoring images from the different reconstructions of the wrist radial-ulnar deviation maneuver based on a 5-point scale (4: strongly agree; 3: agree; 2: neutral; 1: disagree; 0: strongly disagree) in response to three clinically-relevant statements:

1. *I can confidently measure the scapholunate (SL) gap*. This statement is of relevance for assessing dynamic SL instability[29];
2. *I can confidently assess the scaphoid translation with respect to distal radius*. This statement is of value to understanding scaphoid-radial motion and maltracking after trauma[30];
3. *I can confidently assess the trapezium translation with respect to the scaphoid*. This statement is of importance in determining dynamic instability associated with the scaphoid-trapezial joint[31].

For the evaluation study, each radiologist independently scored a total of 120 real-time image volumes (10 right wrists x 4 reconstructions (gridding, FD-sparsity, DCT-sparsity, and FD+DCT sparsity) x 3 sampling schemes (100, 60, and 40 spokes)). They were blinded to the type of reconstruction and sampling scheme, and the real-time 3D datasets (stacked 2D slices) were presented in a random fashion, allowing them to choose slices from which they could evaluate the SL gap, scaphoid translation and trapezium translation. The overall performance of each reconstruction scheme was quantified as the scores from one question for a given reconstruction method, averaged (mean and standard deviation) across different number of spokes. The inter- and intra-rater reliability was estimated using Fleiss’ and Cohen’s kappa (*κ*) coefficient, respectively. The intra-rater reliability was evaluated by asking one of the radiologists to repeat the scoring of 36 randomly chosen images (four subjects). The interval between the two scoring sessions was about a month. Additional measures of image quality were obtained in a test-retest setting. For this, Bland-Altman analysis was performed to quantify test-retest capability of the radial-fast-GRE sequence for varying number of spokes. The plots were computed based on SNR estimated from two measurements chosen randomly from the three repeated measurements. SNR was defined as the ratio of mean signal intensity in the region of interest (ROI) containing the carpal bones to the standard deviation of the background signal. The x- and y-axes of the Bland-Altman analysis were defined as the average and difference, respectively, in SNR at a single time point for the first and second repeat respectively during radial-ulnar deviation maneuver for each participant.

### Scapholunate (SL) gap measurement

To demonstrate the application of the proposed real-time MRI acquisition for providing quantitative measures of commonly presented SL injuries, we measured the SL gap through the middle of the SL articulation on coronal sections for all ten subjects’ right wrists during the radial-ulnar deviation and clenched fist maneuvers. This SL gap was defined as the distance between the cortices of the scaphoid and lunate, at the half-way point between the Gilula lines of the midcarpal and radiocarpal joints[11, 32]. A customized MATLAB graphical user interface was used. Additionally, the SL gap was measured on the high-resolution, static T_1_-weighted images. All SL gap measurements were independently measured by two expert observers, and the inter-observer agreement was measured.

## Results

### Comparison of bSSFP and fast-GRE acquisitions

**Fig 1:**
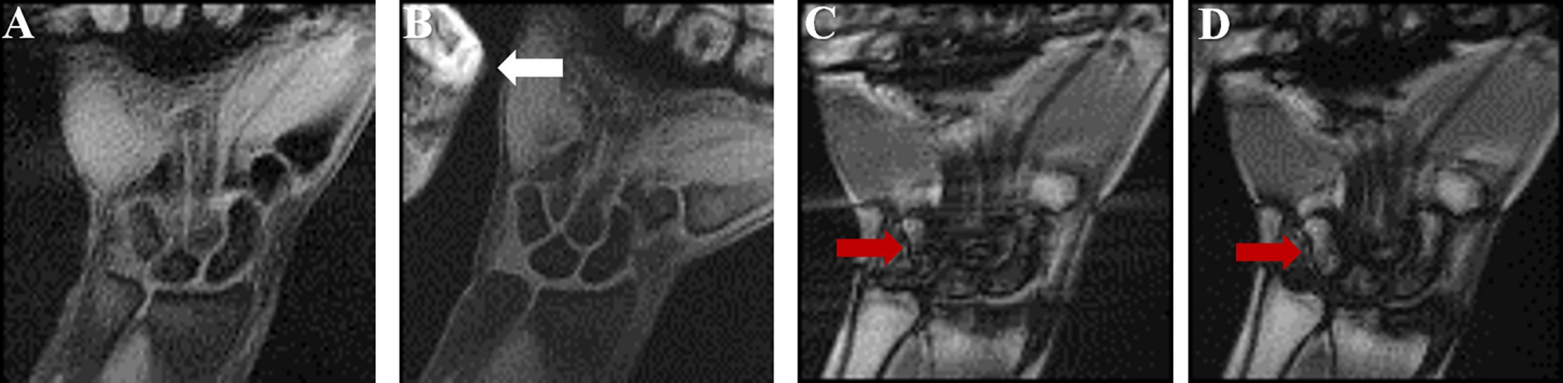
Comparison of real-time MRI pulse sequences, namely (a) radial-fast GRE, (b) Cartesian-fast GRE, (c) radial-bSSFP, and (d) Cartesian-bSSFP from study participants during radial-ulnar deviation. White and red arrows point to the wraparound effect and banding artifacts, respectively.

**Fig 2:**
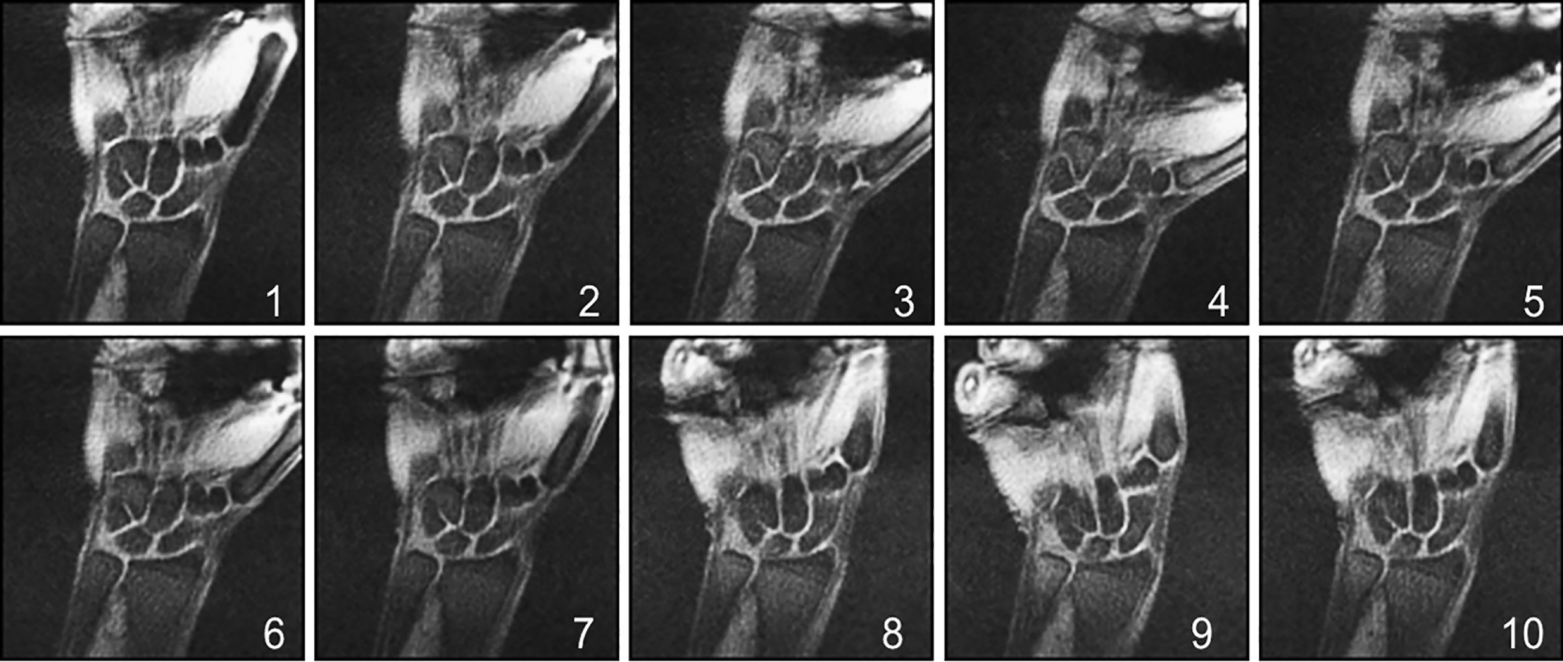
Representative coronal section through the wrist at the various time-points during the radial-ulnar deviation maneuver, with spokes = 100.

Fig 1 shows coronal slices for comparing the real-time MRI pulse sequences during the wrist radial-ulnar deviation maneuver. In each case, the number of spokes or Cartesian lines in k-space were the same (100 lines) and images were reconstructed by the gridding-method. Fig 1 also indicates the wrap-around artifact, which is an inherent artifact associated with Cartesian sampling, and the characteristic banding artifacts arising from the bSSFP pulse sequence[11]. Radial sampling was found to be more robust compared to Cartesian sampling when dealing with these artifacts. Based on these assessments, the radially-sampled-fast-GRE approach was optimized further. A representative coronal section acquired at each of the 10 time points during the wrist’s continuous radial-ulnar deviation maneuver is shown in Fig 2.

### Temporal resolution in radial-fast-GRE

**Fig 3:**
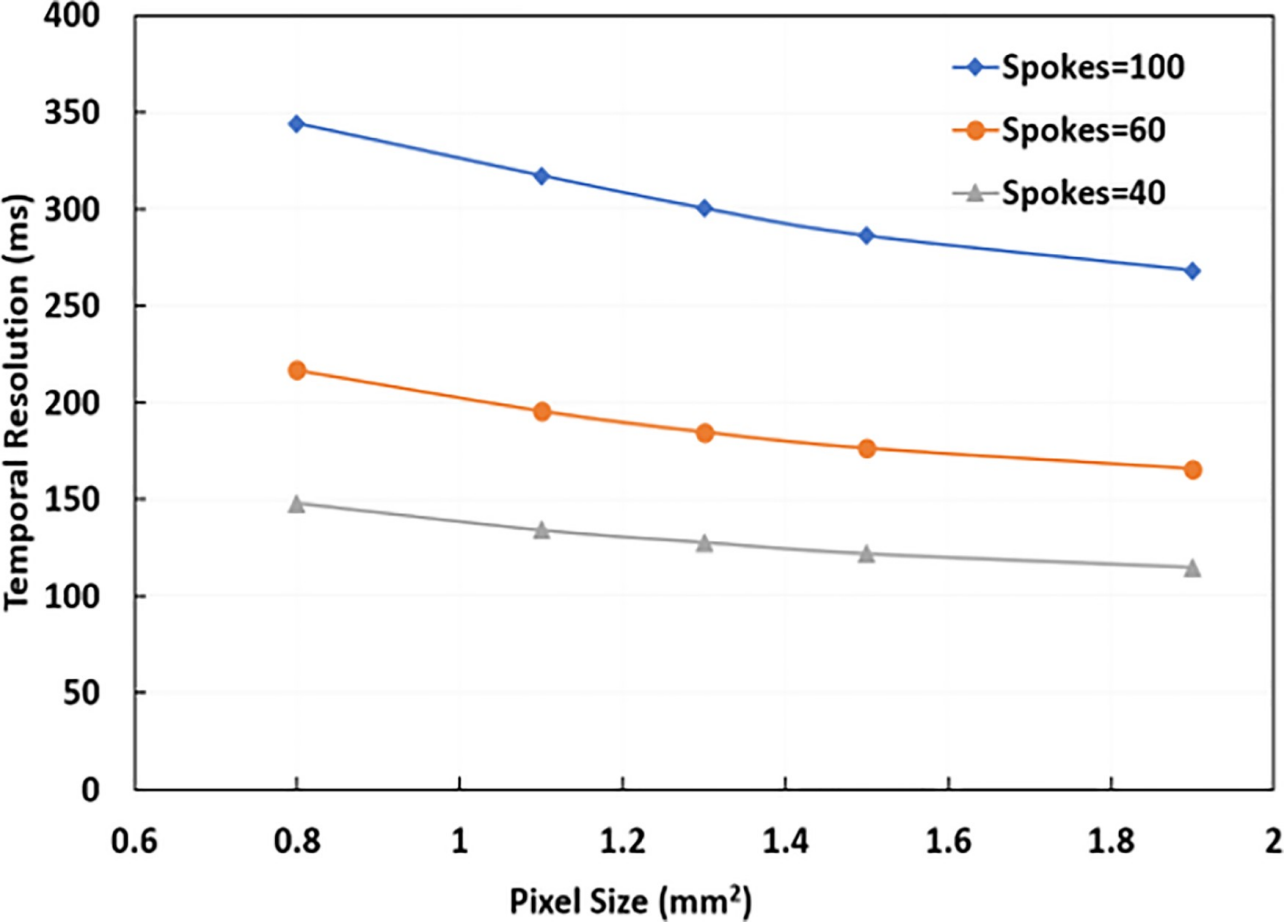
Illustration of different temporal resolutions and pixel sizes achievable with the radial-fast GRE sequence given in Table 1. The graph also describes the trade-off between the number of spokes and temporal resolution.

Fig 3 illustrates the trade-off between temporal resolution, number of spokes, and pixel size (in-plane resolution) for the radial-fast-GRE pulse sequence. Reducing the number of spokes (angular undersampling) resulted in a higher temporal resolution compared to just increasing the pixel size. These assessments converged on a pixel size of 1.07 x 1.07 mm and the parameters in Table 1, as a trade-off between SNR and joint space delineation.

### Constrained image reconstruction

**Fig 4:**
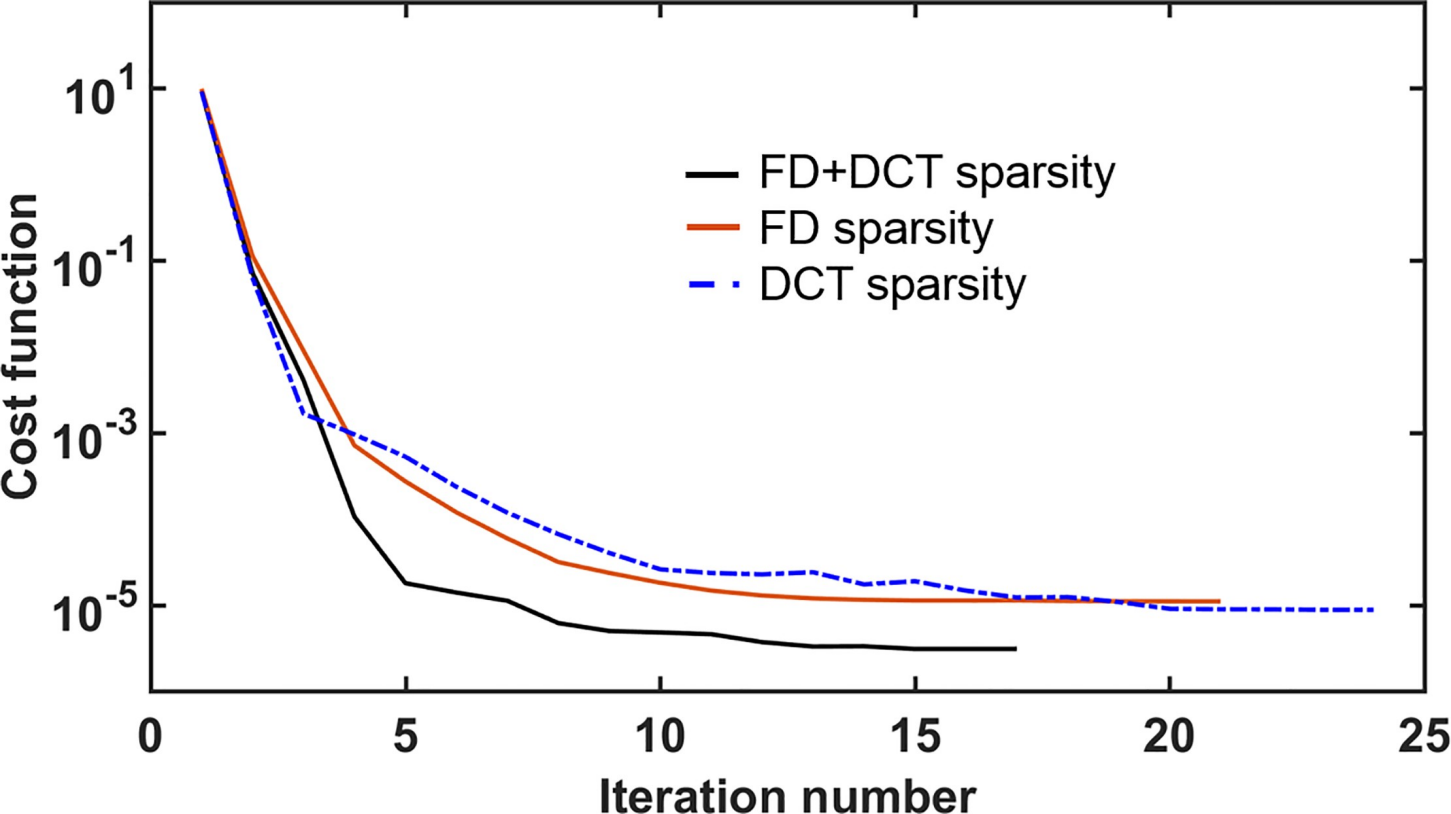
Graph illustrating the convergence curves for FD+DCT sparsity, FD-sparsity, and DCT-sparsity-based penalties for the case of 40 spokes in Fig 5.

**Fig 5:**
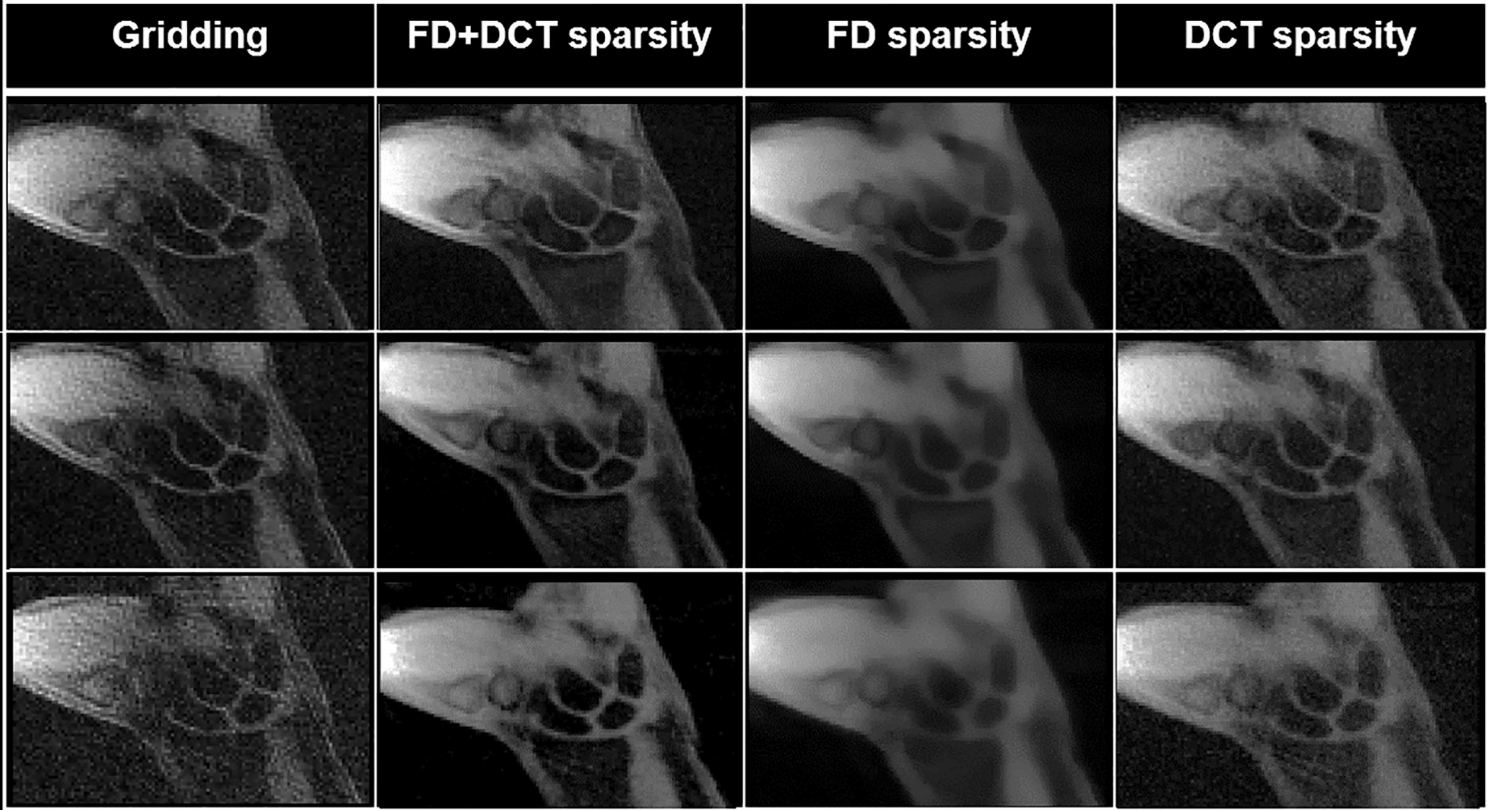
Representative coronal slices of a participant’s right wrist at a time point during radial-ulnar deviation obtained from gridding, FD+DCT sparsity, FD-sparsity, and DCT-sparsity-based reconstruction schemes using 100, 60, and 40 spokes.

The best regularization parameters estimated through L-curves were used for DCT-sparsity and FD-sparsity-based reconstruction, as the values at the maximum curvature (independently for 100, 60, and 40 spokes). For the FD+DCT-based sparsity penalty, the regularization parameters were empirically determined such that the range of *α* was found to be [1×10^−5^: 1×10^−6^] and *β* was found to be [1×10^−6^: 1×10^−8^]. These values provided a fair balance between priors (for spokes = 100, 60, and 40). In addition, we found that with higher undersampling, higher values of *α* and *β* were required to reduce the undersampling artifacts. Fig 4 compares the mathematical convergence curves of DCT-sparsity, FD-sparsity and FD+DCT-sparsity-based penalties (corresponding to spokes = 40 from Fig 5), our fastest method for data acquisition. The curves show the number of iterations required by each type of penalty to reach an empirically chosen stopping criterion of 1×10^−6^ (i.e., when the relative error of the cost function was less than 1×10^−6^). Note that the FD+DCT-sparsity penalty converged faster than the other two penalties indicating the improvement via using multiple priors in the cost function (Eq (1)).

**Fig 6:**
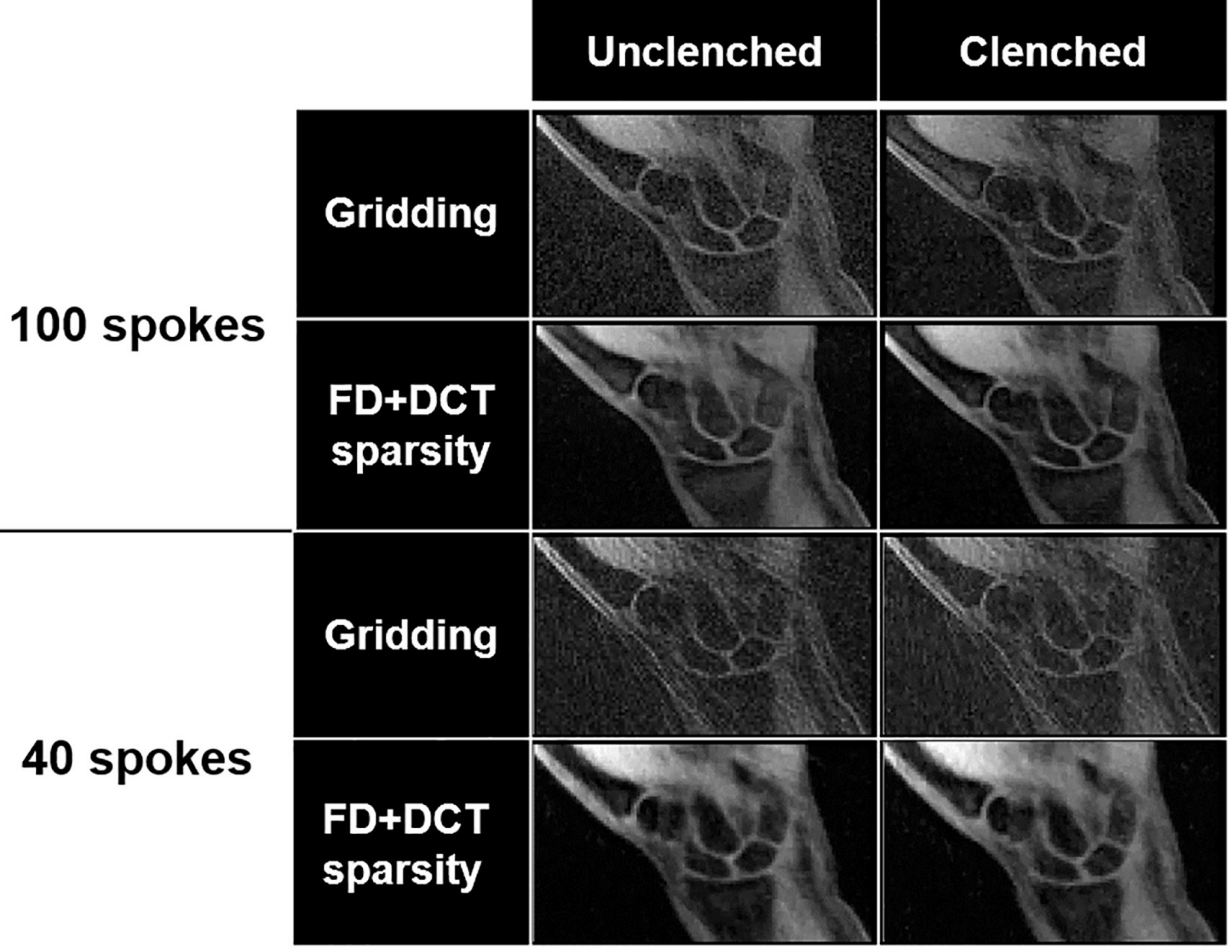
The same participant’s right wrist as shown in Fig 5 at a time point during clenched-fist maneuver using the gridding and FD+DCT sparsity reconstruction schemes using 100 and 40 spokes.

The reconstructed coronal slices corresponding to a single time point from the reconstruction schemes during radial-ulnar deviation and clenched-fist maneuvers of the wrist are shown in Figs 5 and 6 respectively. Comparison of the different reconstruction schemes revealed that all iterative reconstruction schemes were effective in reducing the overall image noise apparent in gridding reconstructions. Although the FD-sparsity-based penalty reduced excessive streaking artifacts, it also induced spatial blurring. This was attributed to the staircasing artifact associated with the first-order penalty and that the regularization parameter estimated may not be optimal for the visual analysis. The DCT-sparsity-based penalty preserved anatomical features, such as edges, but had residual streaks in the reconstruction (evident with spokes = 40). These artifacts were mitigated by simultaneous incorporation of FD+DCT-sparsity-based penalties, especially with a lower number of spokes (60 and 40), that rendered much sharper images. Representative movies showing radial-ulnar deviation (Fig 5) and clenched-fist maneuvers (Fig 6) are available as S1 and S2 Movies. Images reconstructed from 100 spokes rendered relatively discontinuous motion while images corresponding to 60 and 40 spokes rendered smoother motion. The sharpness for feature edges obtained with FD+DCT-sparsity-based reconstruction showed that constrained reconstruction can improve the visualization of the joint spaces in the wrist particularly with 60 and 40 spokes. The FD+DCT-sparsity-based penalty offered an overall better visualization of the joint spaces compared to all other methods evaluated.

### Image scoring by radiologists

The averaged (mean ± standard deviation) visual scores assigned by the three radiologists for all the reconstruction schemes are given in Table 2. The average scores suggest that the FD+DCT-sparsity-based reconstruction scheme consistently outperformed other schemes for all three clinical questions. The inter-rater correlation coefficient (*κ*) ranged between 0.41 and 0.77 for the various reconstruction and spokes (highest for FD+DT sparsity, indicating substantial intra-rater reliability). Additionally, the intra-rater correlation coefficient (Cohen’s *κ*) was found to be 0.83 for the FD+DCT sparsity method. Selecting the slice for scoring was found to be highly repeatable, as there were only 6 coronal slices per timepoint, each with a slice thickness of 6 mm.

**Table 2:** Average image scores of ten subjects’ datasets for all four reconstruction schemes by the three radiologists (R1, R2, and R3) for the clinically-relevant questions. Values given are mean ± standard deviation (SD) of radiologist-assigned image quality scores. The statements were: S1: I can confidently measure the SL gap; S2: I can confidently measure scaphoid translation with respect to distal radius; S3: I can confidently measure trapezium translation with respect to the scaphoid. The 5-point rating scale was 0: strongly disagree; 1: disagree; 2: neutral; 3: agree; 4: strongly agree.

**Clinical Score**	**Gridding**			**FD+DCT sparsity**		
	R1	R2	R3	R1	R2	R3
S1	1.3±1.0	0.6±0.6	1±1.0	3.0±0.9	1.7±1.0	3.2±0.9
S2	1.6±1.0	0.6±0.7	1.5±1.0	3.2±0.9	1.9±0.9	3.3±0.4
S3	2.0±1.3	0.7±0.7	1.9±1.0	3.1±1.0	1.8±1.0	3.4±0.4
	**DCT sparsity**			**FD sparsity**		
	R1	R2	R3	R1	R2	R3
S1	2.8±1.0	1.4±0.7	2.0±1.0	2.7±0.9	1.2±0.8	3.0±0.6
S2	3.0±1.0	1.8±0.9	2.8±1.0	3.2±0.9	1.4±1.0	3.0±0.7
S3	3.1±0.9	1.6±1.0	3.0±0.7	3.1±1.0	1.0±0.8	3.2±0.9

### Test-retest analysis

**Fig 7:**
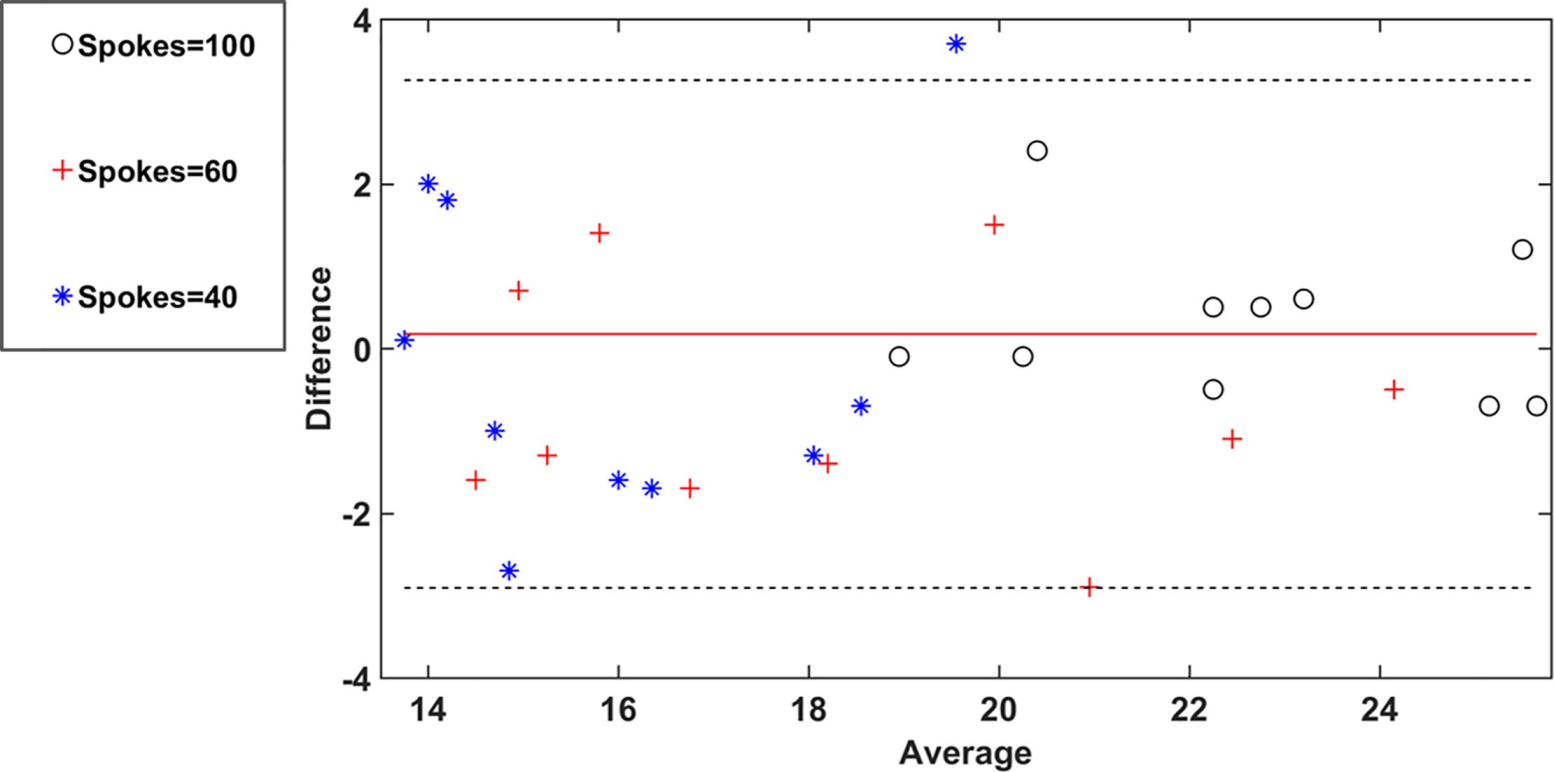
The Bland-Altman plot to quantify test-retest capability of radial-fast GRE sequence for spokes = 100, 60, and 40. The y- and x-axes indicate the difference in SNR between first and second repeat and average of the SNR’s between first and second repeat respectively during radial-ulnar deviation for all the ten subjects’ at a time point. Values from only the right wrist are shown for clarity. The red line indicates the mean of the differences, while the 95% confidence intervals are denoted by the pair of dotted black lines.

Bland-Altman analysis used to quantify test-retest capability of radial-fast GRE sequence for spokes = 100, 60, and 40 is shown in Fig 7, based on scans of all ten subjects. The plot shows that most of the SNR points lie within the 95% confidence interval of the mean of the differences, quantifying the robustness of radial-fast-GRE sequence. Also note the decreasing SNR values on the x-axis with spokes = 100, 60, and 40, suggesting that reducing the spokes from 100 to 40 reduces SNR by almost half, as would be expected.

### SL gap measurement

**Fig 8:**
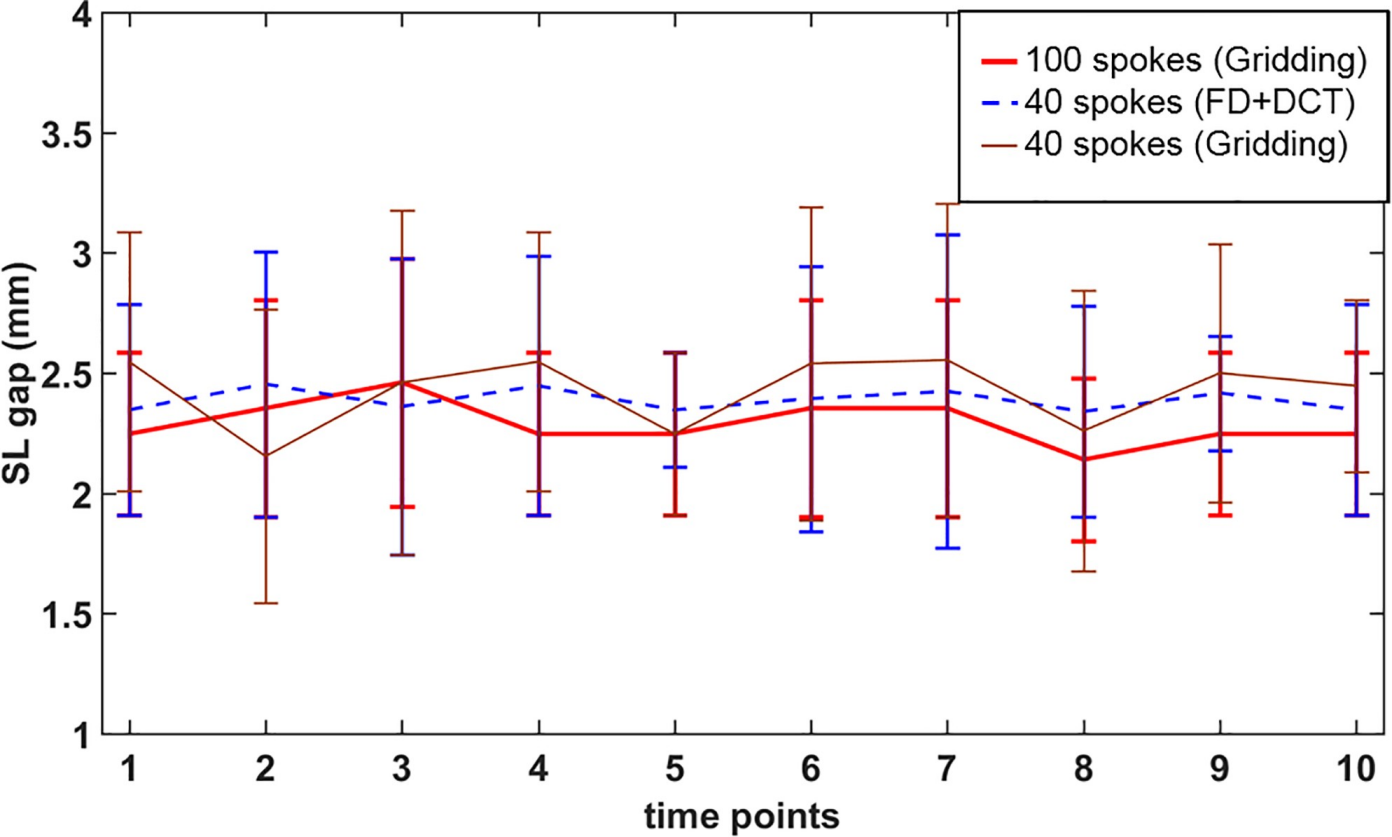
The SL gap (mean ± standard deviation (SD)) during radial-ulnar deviation maneuver at every time point measured by tracking a single slice through the right wrist for the 10 subjects. The measurements were obtained from gridding-based reconstruction for 100 and 40 spokes, and FD+DCT sparsity-based reconstruction for 40 spokes. The error bars indicate the SL gap variation across the 10 subjects at each time point.

The SL gap of 1.64±0.63 mm was measured for the high-resolution, static T_1_-weighted images. These values are within the ranges reported in the literature[11, 33]. The SL gaps for 100 spokes with gridding, 40 spokes with gridding, and 40 spokes with FD+DCT-sparsity-based reconstruction were 1.52±0.67 mm, 1.79±1.01 mm and 2.10±0.93 mm, respectively. Fig 8 shows a comparison between SL gap measurements for the 100 and 40 spokes cases, relative to the static T_1_-weighted images, at the ulnar-deviation, neutral, and radial deviation, while Table 3 shows quantitative values for clenched-fist maneuver. The higher SL gap from images reconstructed from 40 spokes and FD+DCT-sparsity compared to the high resolution static T_1_-weighted images (mean difference of 0.46 mm, less than one half of a pixel width) was attributed to the selection of the regularization parameters (particularly for the FD-component) that appeared to prefer image smoothness to spatial resolution. The values obtained however are within the range of SL gap values reported in the literature for wrists without pathology[11, 33]. The SL gap widened, albeit insignificantly, as the wrist went from the relaxed to the clenched-fist position, consistent with the literature[11, 34]. For reference, an SL gap of greater than 3 mm is typically indicative of scapholunate instability or dissociation[35, 36]. The inter-rater correlation coefficients (*κ*) from the static T_1_-weighted images, 100 spokes with gridding, 40 spokes with gridding, and 40 spokes with FD+DCT sparsity-based reconstruction were 0.78, 0.58, 0.59, and 0.75 (p<0.001). The inter-rater correlation coefficient of the FD-DCT sparsity was closest to that from high-resolution, static T_1_-weighted images, attributed to the reduction of streaking artifacts and noise due to undersampling.

**Table 3:** SL gap measurement during the clench-unclench maneuver for all ten subjects. Values are given as mean±SD across the subjects.

Metric	Unclenched fist			Clenched fist		
	Gridding Spokes = 100	FD+DCT Spokes = 40	Gridding Spokes = 40	Gridding Spokes = 100	FD+DCT Spokes = 40	Gridding Spokes = 40
SL gap (mm)	2.3±0.3	2.4±0.5	2.5±0.5	2.4±0.5	2.5±0.7	2.3±0.6

## Discussion

Clinical MRI protocols are currently limited to assessing the wrist in its static position. However, dynamic carpal instability is manifested and diagnosed during normal wrist motion or with loading. In Boutin et. al.[11], the achievable temporal resolution of dynamic carpal imaging was about 475–562 ms per slice using the 2D bSSFP sequence. Additionally, since bSSFP is susceptible to banding artifacts, a dielectric pad was attached to the wrist to reduce the magnetic field inhomogeneity, but it had the undesired effect of limiting the range of motion[11]. The proposed fast-GRE sequence, which utilizes shorter TE and TR, is less sensitive to tissue susceptibility differences, making it more suitable for real-time imaging. A radial-sampling scheme was used, instead of Cartesian sampling, as the former is less sensitive to motion artifact/temporal blurring due to inherent averaging of low spatial frequencies[37]. This work shows an approximately 4-fold increase in temporal resolution compared to existing methods, i.e. 4 slices (either in space or time) could be acquired in the time required for acquiring data for a single slice with previous methods[11, 16].

The radiologist-provided image quality scores suggest that FD+DCT-sparsity-based penalty significantly improved the reconstructed image quality (by about two-fold) and inter-rater reliability, in comparison with the gridding method with the same number of spokes. The use of FD+DCT-sparsity was shown to have fewer overall artifacts due to undersampling compared to single priors such as FD or DCT, consistent with other applications[38, 39]. On the other hand, the FD+DCT-sparsity images showed a slightly higher SL gap than the high-resolution static T_1_-weighted images (difference was about half the width of one pixel), although the inter-rater correlation coefficients were comparable between the two, and significantly better than those obtained from gridding and other schemes. These characteristics are attributed to a trade-off between image smoothness and spatial resolution, induced by the selection of the penalty functions and regularization parameters, when acquiring the images at a high temporal rate. Understanding this trade-off is critical for determining and optimizing the future clinical role of real-time MRI for examining suspected dynamic carpal instability. To the best of our knowledge, this is the first work that has demonstrated the use of radial-fast-GRE sequence with constrained reconstruction to evaluate the moving wrist. In addition to SL gap assessments, our image quality assessments also involved the evaluation scaphoid translation with respect to the distal radius and trapezium from real-time MRI. Abnormal translation of the scaphoid with respect to the distal radius is considered to reflect ligamentous injury[30, 40] and with the effective identification of such abnormal trajectories of the scaphoid from real-time MRI, it may be possible to address malalignment and prevent the development of arthritis at the joint. The scaphoid-trapezial joint is a common site of osteoarthritis[31], and identifying dynamic instability at the joint from real-time MRI may be helpful in choosing appropriate treatments for the condition[41].

The image scores assigned by reviewers 1 and 3 were higher than those provided by reviewer 2, although the scores assigned by all reviewers improved in a consistent manner for the proposed method compared to gridding. There are intrinsic differences in the manner in which a radiologist may score images, such as their personal emphasis on different image characteristics, such as crisp edges, overall SNR, or image smoothness, and this variability is routinely encountered for evaluating images from a new modality[42, 43], as is the case with the proposed real-time MRI. Development of a real-time MRI-specific training regimen within a musculoskeletal radiology subspecialty training program in the future may be helpful in reducing inter-rater differences. Furthermore, for optimizing imaging parameters, such as voxel size and bandwidth further for improving the assessment of the SL gap, studies in dynamic phantoms that realistically reproduce wrist motion and the associated magnetic susceptibility may be useful.

If a wrist coil is used in routine clinical practice at the imaging site implementing real-time MRI, it is possible that a coil change may be needed (to a head or knee coil) that can accommodate maneuvers involving larger ranges of wrist motion. Based on our experience, this may add less than 8 min to the typical 30–45 wrist scan (this includes coil change, landmarking, set up and real-time MRI acquisition). If a coil change is not necessary, such as for the clenched fist maneuver, the time overhead for real-time MRI acquisition is just 1–2 min. Subject training, focusing primarily on providing guidance regarding the speed at which the maneuver must be performed, may take of the order of 2 min.

Dynamic computed tomography (CT) has been used successfully to investigate the normal kinematics and pathokinematics of the wrist[44, 45] and to provide measures for assessing dynamic carpal instability[12]. However, CT involves ionization radiation, has poor soft tissue contrast compared to MRI, and generally is ordered as a separate exam and hospital visit. Standard MRI or MRI arthrography does not consistently diagnose all ligamentous tears[7, 46] and may show asymptomatic ligament defects that may have minimal clinical (or biomechanical) significance[47]. Thus, a real-time MRI exam could provide currently unavailable knowledge regarding wrist instability, and furthermore, show any clunk or sudden change in intercarpal alignment[48–50]. The real-time MRI exam is feasible as a supplement to a standard static MRI exam. A comparison of real-time MRI with dynamic CT or MRI arthrography however was outside the scope of this work but will be pursued in the future.

Our study had some limitations. First, the constrained, iterative reconstruction scheme was computationally intensive compared to gridding. The average time for FD+DCT-sparsity reconstruction was about 80 s per slice, while gridding required only 15 s. This limitation can be addressed in the future by using high-performance graphics processing units (GPUs)[44]. Second, since the wrist images are not inherently sparse in their pixel representation, we used DCT as the sparsifying transform. Wavelets, such as Daubechies 4, have the restricted isometry property that may make them more suited for real-time MRI[18]. A detailed comparison of DCT versus wavelets will be conducted in future work. Third, although radiologists’ ratings and SL gap were used as measures to compare reconstruction methods in this paper, we acknowledge that measures such as contrast-to-noise ratio may be useful in evaluating other properties of the reconstructed images. Fourth, the regularization scheme was applied only in the spatial dimension and not along the temporal dimension. Application of temporal regularization schemes could further provide improved reconstruction quality with fewer radial spokes (< 40), thereby improving the temporal resolution[51, 52]. In addition, non-Cartesian sampling schemes suitable for motion imaging, such as golden-angle and spiral sampling, and image reconstruction methods utilizing machine learning approaches will be explored in our future work. These strategies may be effective to reconstruct artifact-free wrist images with a higher spatial and temporal resolution, allowing more subtle SL gap differences to be measure with a higher accuracy.

## Conclusions

We demonstrated the feasibility of a real-time MRI scan protocol with a temporal resolution of up to 135 ms per slice (~7 fps) for assessing continuous, active, and uninterrupted wrist motion, and to extract anatomical information relevant to evaluating dynamic carpal instability. The proposed real-time protocol utilizes a short scan time, typically less than 20 s per maneuver, and was deemed suitable as a supplement to a standard clinical wrist MRI exam with minimal disruptions to clinical workflow. The proposed technique could represent a paradigm shift for clinical MRI, further our understanding of baseline patterns of carpal kinematics and improve our ability to assess dynamic carpal instability.

## Supporting information

S1 Movie Fly-through from the real-time MRI in time and space (slices) during the performance of the radial-ulnar deviation maneuver for FD+DCT-based constrained reconstruction with spokes = 40. (AVI)

S2 Movie Fly-through from the real-time MRI in time and space (slices) during the performance of the clenched-fist maneuver for FD+DCT-based constrained reconstruction with spokes = 40. (AVI)
